# Molecular epidemiology of the first wave of severe acute respiratory syndrome coronavirus 2 infection in Thailand in 2020

**DOI:** 10.1038/s41598-020-73554-7

**Published:** 2020-10-06

**Authors:** Jiratchaya Puenpa, Kamol Suwannakarn, Jira Chansaenroj, Pornjarim Nilyanimit, Ritthideach Yorsaeng, Chompoonut Auphimai, Rungrueng Kitphati, Anek Mungaomklang, Amornmas Kongklieng, Chintana Chirathaworn, Nasamon Wanlapakorn, Yong Poovorawan

**Affiliations:** 1 Center of Excellence in Clinical Virology, Department of Pediatrics, Faculty of Medicine, Chulalongkorn University, Bangkok, Thailand; 2 Department of Microbiology, Faculty of Medicine, Siriraj Hospital, Mahidol University, Bangkok, Thailand; 3 Institute for Urban Disease Control and Prevention, Department of Disease Control, Ministry of Public Health, Bangkok, Thailand; 4 Department of Microbiology, Faculty of Medicine, Chulalongkorn University, Bangkok, Thailand; 5 Division of Academic Affairs, Faculty of Medicine, Chulalongkorn University, Bangkok, Thailand

**Keywords:** Microbiology, Virology, SARS-CoV-2

## Abstract

The coronavirus disease 2019 pandemic caused by severe acute respiratory syndrome coronavirus 2 (SARS-CoV-2) is a major global concern. Several SARS-CoV-2 gene mutations have been reported. In the current study associations between SARS-CoV-2 gene variation and exposure history during the first wave of the outbreak in Thailand between January and May 2020 were investigated. Forty samples were collected at different time points during the outbreak, and parts of the SARS-CoV-2 genome sequence were used to assess genomic variation patterns. The phylogenetics of the 40 samples were clustered into L, GH, GR, O and T types. T types were predominant in Bangkok during the first local outbreak centered at a boxing stadium and entertainment venues in March 2020. Imported cases were infected with various types, including L, GH, GR and O. In southern Thailand introductions of different genotypes were identified at different times. No clinical parameters were significantly associated with differences in genotype. The results indicated local transmission (type T, Spike protein (A829T)) and imported cases (types L, GH, GR and O) during the first wave in Thailand. Genetic and epidemiological data may contribute to national policy formulation, transmission tracking and the implementation of measures to control viral spread.

## Introduction

In December 2019 patients presenting with viral pneumonia of unknown cause were reported in Wuhan, China. In January 2020 a novel human coronavirus provisionally named ‘2019 novel coronavirus’ was identified via next-generation sequencing^1,2^. The International Committee on Taxonomy of Viruses subsequently changed the official name of the virus to ‘severe acute respiratory syndrome coronavirus 2′ (SARS-CoV-2)^3^, and the disease it caused was dubbed ‘coronavirus disease 2019′ (COVID-19). On 30 January 2020 the World Health Organization declared the SARS-CoV-2 outbreak a so-called ‘Public Health Emergency of International Concern’, and on 11 March 2020 it declared the outbreak a pandemic^4^. As of 31 May 2020 more than 6 million people had been infected with SARS-CoV-2, and there had been more than 373,000 deaths^5^.

SARS-CoV-2 belongs to the family *Coronaviridae*, the subfamily *Coronavirinae*, and the order *Nidovirales*. The genome of coronaviruses consists of positive-stranded RNA of approximately 27 to 32 kb in length, including 7 to 10 open reading frames (ORFs) and untranslated regions at the 5′ and 3′ ends of the RNA^6^. Based on their genomic diversity coronaviruses are divided into four genera; alphacoronaviruses, betacoronaviruses, gammacoronaviruses, and deltacoronaviruses. Prior to the emergence of SARS-CoV-2, in the last two decades six other coronaviruses have been detected in humans. Two are alphacoronaviruses (human coronavirus NL63 and human coronavirus 229E) that usually cause mild upper respiratory disease^7^. The other four are betacoronaviruses, including the weakly pathogenic human coronavirus OC43 and human coronavirus HKU1, and the highly pathogenic severe acute respiratory syndrome coronavirus (SARS-CoV) and Middle East respiratory syndrome coronavirus (MERS-CoV). SARS-CoV-2 is the seventh human betacoronavirus, and its genome is closely related to SARS-CoV which emerged in 2002 and 2003^1^.

As of 30 May 2020 there were more than 36,000 SARS-CoV-2 genome sequences in the GISAID database (https://www.gisaid.org/), contributed by numerous laboratories around the world. During the early period of the outbreak genome sequencing revealed two types of viruses based on differences in two single nucleotide polymorphisms in ORF1ab and ORF8; L type and S type^8^. Another analysis categorized SARS-CoV-2 into three types, A, B, and C, based on amino acid changes^9^. As of 30 May 2020 there were seven clades identified in the GISAID database, S, L, V, G, GR, GH, and O. SARS-CoV-2 genetic variation may be associated with differences in viral replication^10^, though more evidence is needed to verify any putative associations between mutations and pathogenesis in humans.

In Thailand the Ministry of Public Health reported the first laboratory-confirmed case of SARS-CoV-2 in a 61-year-old Chinese traveller who had arrived from Wuhan on 12 January 2020. This was reportedly the first recorded case outside of China^11^. By the end of January two confirmed cases had been reported, both Thai nationals. Of those, one was a 73-year-old woman from Nakhon Pathom Province who had recently returned from China. The first unequivocally domestically contracted SARS-CoV-2 infection in Thailand was documented on 31 January 2020 when a taxi driver who had not travelled outside Thailand tested positive. In January the percentage of confirmed cases in which the patients were travellers from other countries was 89.5% (17/19), but the corresponding percentage in February was 65.2% (15/23). On 29 February 2020 SARS-CoV-2 was designated a dangerous communicable disease under the Communicable Disease Act. The Act stipulates that all infected people must be hospitalized. On 15 March 2020 COVID-19 spread within a boxing stadium and drinking venues in Bangkok, then it spread throughout Thailand^12^. Notably however, most cases confirmed in April were people who had returned from a mass religious meeting in Indonesia, Thai workers from Malaysia, and immigrants at the detention centre in Songkla province^13,14^.

Similar to China, the incidence of COVID-19 in Thailand decreased dramatically when the Thai government prohibited social gatherings after the first wave of the rapid spread of SARS-CoV-2. On 16 March 2020 the Thai government announced that the Thai New Year’s national holiday (Songkran) between the 13^th^ and 15^th^ of April 2020 would be postponed indefinitely^15^. On 18 March 2020 the Thai government began implementing a social distancing policy, including the mandatory closure of all schools and universities, entertainment and sporting venues, and all stores except food markets^16^. Subsequently the Thai government announced that a nationwide 10 p.m. to 4 a.m. curfew would commence on 02 April 2020. As of 6 June 2020 there had been 3,104 hospitalizations and 58 deaths in Thailand, equating to a fatality rate of < 2%^17^.

In the current study the genotypes of the SARS-CoV-2 strains involved in the outbreak in Thailand during the first wave from February to April 2020 were investigated. Based on the genome sequences available in GIASID, nucleotide variation in four regions of the SARS-CoV-2 genome was used to conduct viral tracking and identify sites of origin of outbreaks in Thailand.

## Methods

### Ethics statement

The research proposal was approved by the institutional review board of the Ethics Committee of the Faculty of Medicine, Chulalongkorn University, Thailand (IRB number 301/63). The institutional review board of the Ethics Committee for human research waived the need for consent because all samples were anonymous. All methods were performed in accordance with the relevant guidelines and regulations.

### Study population and sample collection

**Figure 1:**
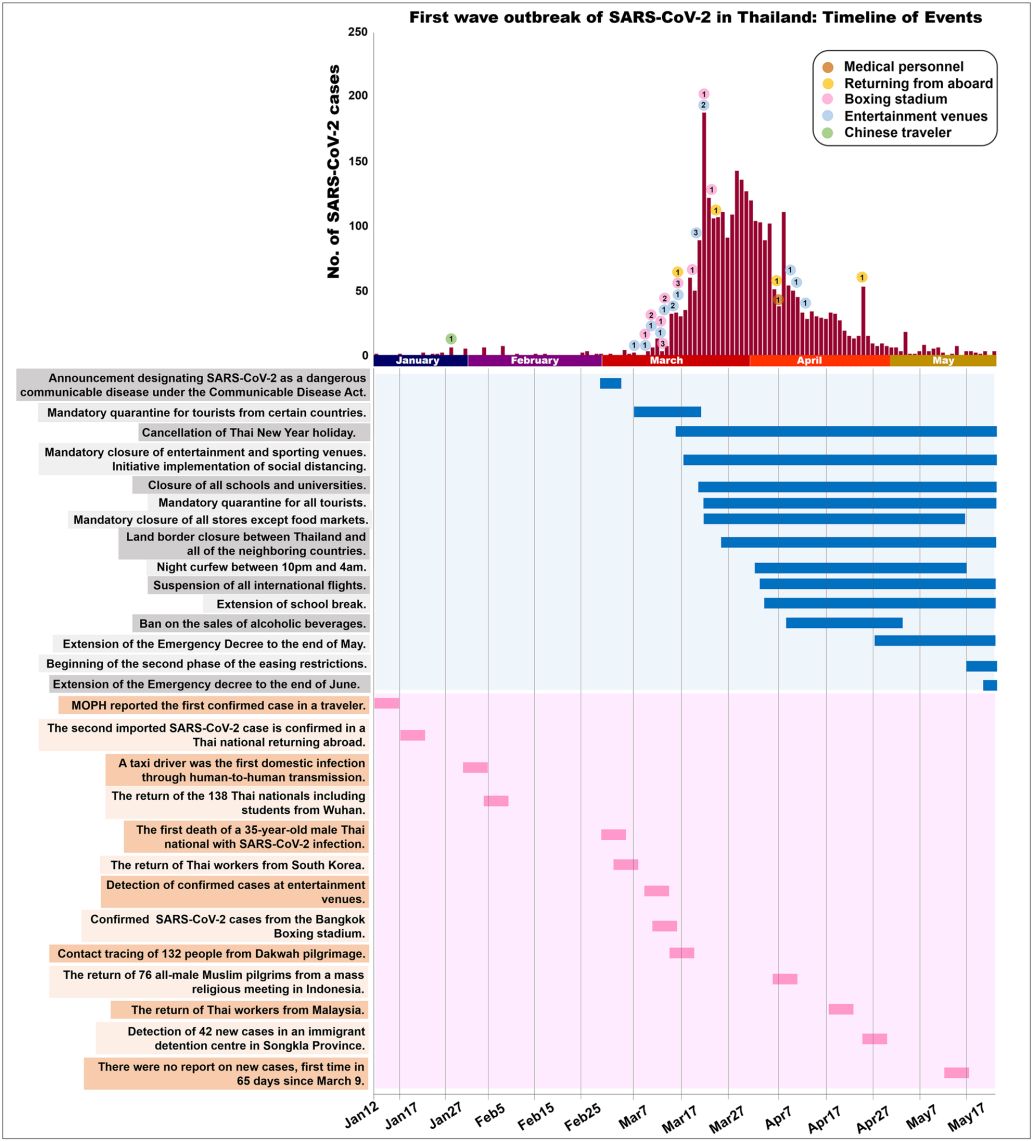
The first wave of SARS-CoV-2 outbreak in Thailand: Timeline of Events and the number of specimens.

The study was conducted using anonymized SARS-CoV-2-positive specimens collected from a diagnostic service except for the first specimens from the Chinese traveller from Wuhan (provided from the Department of Medical Science, Ministry of Public Health, Thailand). Patient identifiers including personal information and hospitalization number were removed from the samples to ensure patient confidentiality. The demographic data recorded included sex, age, vital signs and exposure history. The specimens were collected from Bangkok, Nonthaburi, Samut Prakan, Songkla, Ubon Ratchathani and Yala (Figure S1). The samples included a record of the date they were procured, and the putative location of infection (boxing stadium, specific drinking venues, a mass religious meeting from Indonesia, Thai workers from Malaysia, and an immigrant detention centre, among others) (Fig. 1).

### RNA extraction and polymerase chain reaction sequencing

A total of 40 positive nasopharyngeal and/or throat swab samples (except the first specimens from the Chinese traveller from Wuhan) were confirmed to be SARS-CoV-2-positive via two separate multiplex real-time reverse transcription polymerase chain reaction (RT-PCR) assays. In the first multiplex assay the Allplex 2019-nCoV (Seegene, Seoul, Republic of Korea) incorporating primers and probes specifically targeting RdRp, N and E genes was used. The second assay used the LightMix Modular SARS and Wuhan CoV (TIB-Molbiol, Berlin, Germany) incorporating primers and probes corresponding to the RdRp and E genes. Viral RNA was extracted from 200 µL of sample using a magLEAD 12gC instrument (Precision System Science, Chiba, Japan) with a magLEAD Consumable Kit (Precision System Science, Chiba, Japan) in accordance with the manufacturer’s instructions. All RNA specimens were transferred to the Center of Excellence in Clinical Virology at Chulalongkorn University for conventional PCR and sequencing, and SARS-CoV-2 RdRp, S, N and E genes were amplified via a primer set specific for SARS-CoV-2 (Table S1).

The one-step RT-PCR reactions were conducted using the SuperScript III Platinum One-Step RT-PCR System with Platinum *Taq* DNA Polymerase (Invitrogen, Carlsbad, CA). Briefly, the PCR reaction mixture contained 2–3 μL of RNA, 0.5 μM of each primer, 12.5 μL of 2X Reaction Mix (Invitrogen) and 1 μL of SSIII RT/Platinum Taq Mix, and was adjusted to a final volume of 25 μL with nuclease-free water. Amplification was conducted in a thermal cycler (Eppendorf, Hamburg, Germany) via a protocol including reverse transcription at 45 °C for 30 min, initial denaturation at 94 °C for 3 min, 40 cycles of 30 s of denaturation at 94 °C, 30 s of primer annealing at 53 °C, 90 s of extension at 68 °C, and further extension for 7 min at 68 °C. PCR products were separated on a 2% agarose gel with a 100-base pair DNA ladder and visualized on an ultraviolet trans-illuminator. PCR products were gel-purified using the HiYield Gel/PCR DNA Fragment Extraction kit (RBC Bioscience Co, Taipei, Taiwan). DNA sequencing was performed by First BASE Laboratories Sdn Bhd, Selangor, Malaysia.

### Phylogenetic analysis

The SeqMan II component of the DNAStar software (v. 6.0) was used for nucleotide sequence assembly. Genome sequences were aligned using ClustalW, implemented via the BioEdit program (v. 7.2.0). The MEGA program (v. 6.06) was used for phylogenetic tree construction, which was performed via the neighbour-joining method with 1,000 bootstrap replicates. Evolutionary distances were calculated using the maximum composite likelihood method. Representative sequences from different areas of the world available in the GenBank and GISAID databases were utilized in phylogenetic analysis. Sequences were deposited in GenBank (Accession no. MT502900-MT503099; Table S2).

### Statistical analysis

Statistical analysis was conducted using the Statistical Package for Social Sciences v. 19.0 (SPSS Inc., Chicago, IL). The chi-square test was used to analyse demographic patient factors, and *p* < 0.05 was deemed to indicate statistical significance.

## Results

### First wave of SARS-CoV-2 outbreak in Thailand

The first wave of SARS-CoV-2 outbreak started in early March 2020 and peaked between the 22th and 29th of March 2020. As the Thai government implemented incremental public health measures to mitigate viral transmission, there was a marked overall decline in the SARS-CoV-2 cases since 20 March 2020 (Fig. 1).

### Patterns of SARS-CoV-2 variation in Thailand

**Figure 2:**
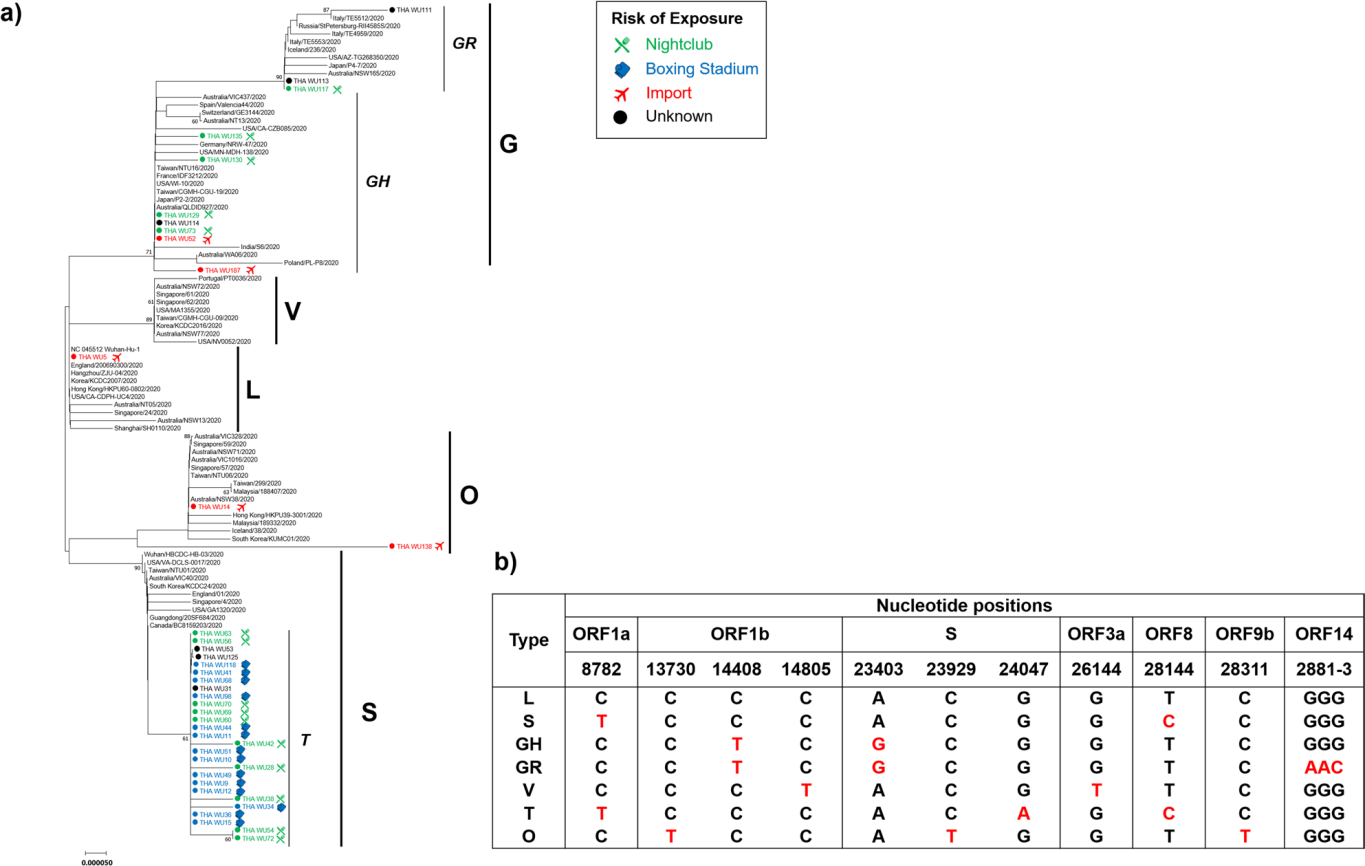
Type of viral variations with exposure history. (**a**) Phylogenetic tree of concatenated sequences, including partial ORF1ab (nucleotide position 8,596–8,927 and 13,259–16,269), S (nucleotide position 21,320–25,541), ORF3a to E (nucleotide position 25,902–26,549), and ORF9b to ORF10 (nucleotide position 28,101–29,682). The phylogenetic tree was generated by the neighbor-joining method with 1,000 bootstrap replicates. Branch values > 60 were indicated. The blanket showed the five main types. Dots and colors precede the sequences isolated in this study with different risks of exposure. b) The pattern of nucleotide substitution change and type of SARS-CoV-2.

The sequences of partial ORF1ab (nucleotides 8,596–8,927 and 13,259–16,269), S (nucleotides 21,320–25,541), ORF3a to E (nucleotides 25,902–26,549), and ORF9b to ORF10 (nucleotides 28,101–29,682) were selected to analyse genetic variations. Phylogenetic analysis of concatenated sequences of worldwide SARS-CoV-2 isolates revealed five main clusters (Fig. 2a). Based on genetic variations and amino acid changes the clusters were defined as L, S, G, V and O types. The types and patterns of nucleotide substitution are shown in Fig. 2b.

Type L originated in China during the first period of the outbreak. Wuhan-Hu-1 (NC_045512) is the reference type L strain. Type S was detected during the early period of the outbreak, and it has nucleotide substitutions at position 8,782 in ORF1ab (C8782T) and 28,144 (T28144C) in ORF8. Types G and V evidently branched off from type L. Type G has single nucleotide changes in ORF1b (C14408T) and S (A23403G). There is also a GR strain that is distinct from the GH strain with a nucleotide substitution at ORF14 (2881–3; GGG to AAC). Type V differed from type L at positions in ORF1b (C14805T) and ORF3a (G26144T). Type O had nucleotide substitutions in ORF1b (C13730T), S (C23929T) and ORF9b (C28311T).

In the present study WU5 was identified as type L, which was closely related to Wuhan–Hu–1. Ten strains were identified as type G, including seven GH strains and three GR strains. Two strains were identified as type O. Most of the samples (27/40) were clustered within the distinct type S branch. This branch was defined as type T based on a nucleotide substitution in the S gene (G24047A) and an amino acid change in the Spike protein (A829T). Therefore, type T was the most prevalent in the samples collected in Thailand in the present study.

### Variation patterns and exposure history

In the current study exposure history was divided into three categories; imported, boxing stadium, and nightclub. ‘Imported’ was defined as any cases in which the patient had returned from an endemic area or had been in contact with travellers who had returned from an endemic area. It also included the aforementioned migrant worker and religious pilgrimage cohorts. ‘Boxing stadium’ was defined as cases in which the patient contracted SARS-CoV-2 from the boxing stadium in Bangkok, or had been in contact with anyone who was most likely infected at that boxing stadium. The boxing stadium group was the largest cluster in the March 2020 outbreak in Bangkok. ‘Nightclub’ was defined as cases in which the patient most likely contracted SARS-CoV-2 from a nightclub, entertainment venue, or restaurant in Bangkok, or had been in contact with a person who was most likely infected at a nightclub.

The WU5 strain was clustered with the L type detected in January 2020 during the first period of the outbreak in Thailand. This isolate was closely related to Wuhan-Hu-1. The likely source of exposure was an imported case from Wuhan, China, the first endemic area. Samples in the imported group also belonged to types GH, GR, and O. Two samples isolated in March 2020 were classified as O types. The travel histories of the patients these samples were derived from indicated that one of them had recently returned from outside of Thailand, and the other was a member of the cohort from the southern part of Thailand who had recently undertaken a religious pilgrimage. The SARS-CoV-2 samples derived from the above-described group of migrant workers in May 2020 were identified as type GR. All samples in the boxing stadium group were identified as type T. These samples were obtained during the large outbreak in March 2020 in Thailand. The SARS-CoV-2 samples derived from the nightclub included GH, GR and T types. Most of the type T samples in the nightclub group were collected in March 2020, and the GH and GR type samples in that group were collected in March and April 2020. Thus, there were multiple SARS-CoV-2 types circulating during this period.

### Viral variation and clinical data

To assess associations between SARS-CoV-2 types and clinical signs, the results of genetic variation analysis were compared with clinical data. Clinical data pertaining to 4 samples was missing, and these samples were excluded from this analysis. By way of this, the one type L sample identified in the present study was excluded from this component of the analysis. Clinical symptoms and SARS-CoV-2 types are shown in Table 1. Most samples were derived from patients with common symptoms of upper respiratory infection such as fever, coughing, and a sore throat. Eight of the patients had pneumonia, and 7 of the samples from these 8 patients were SARS-CoV-2 type T. One patient with type T SARS-CoV-2 required admission to the intensive care unit (ICU). One patient reported diarrhoea, and none of the patients died. There were no significant associations between SARS-CoV-2 type and clinical symptoms.

**Table 1:** Clinical symptoms and the type of viral variation.

	Total (N = 36)	Type				*p* value
		GH (N = 7)	GR (N = 2)	O (N = 1)	T (N = 26)	
Age (average age)	34.62	27.6	25.5	59	36.04	
Gender (Male/Female)	21/15	0/7	1/1	1/0	19/7	
Symptomatic/Asymptomatic	23/13	5/2	1/1	1/0	16/10	0.809
Fever	11	1	1	1	8	0.320
ICU	1	0	0	0	1	0.933
Cough	12	3	1	1	7	0.331
Sore throat	10	2	1	1	6	0.334
Malaise	9	0	1	1	7	0.133
Runny nose	8	1	1	1	5	0.209
Productive cough	7	1	0	0	6	0.814
Headache	6	2	1	0	3	0.333
Diarrhea	1	0	0	0	1	0.949
Anosmia	1	0	0	0	1	0.949
Pneumonia	8	1	0	0	7	0.624

There were four samples of which no clinical data were available. The missing data were excluded from this table.

## Discussion

Viral genome sequencing data have been used to investigate viral transmission and factors associated with it, in a field known as ‘genomic epidemiology’^18^. Several reports describe the use of genomic data to track viral transmission^19–21^. Many reports have described SARS-CoV-2 genome variation and the use of complete genome data to track its transmission^8,9,22^. However, analysis of polymorphic viral genome segment may be helpful in rapidly identifying patterns of epidemiology and viral genetic cluster. In the current study four regions of the SARS-CoV-2 genome were sequenced to identify genetic variants.

In January and February 2020 the confirmed SARS-CoV-2 cases were identified as having been imported from China. Genetic variations of L and S types were identified during the early period of the outbreak in China^8^. One sample in the current study collected in January 2020 was closely related to the SARS-CoV-2 strain circulating in China at that time identified as type L.

The first outbreak in Thailand was evidently associated with a boxing stadium and entertainment venues in Bangkok during March 2020^12^. All the samples associated with that outbreak analysed in the present study were type T. Type T branched off from type S, which originated from China, but type T has not been identified in other countries. This indicated local transmission in Bangkok. Interestingly, after the first outbreak in March 2020 type T was detected less frequently. This may have been a result of intervention policies such as mandatory closure of sporting and entertainment venues (Fig. 1).

The mandatory closure of public places may help to control local transmission. Notably however, there were several cases of patients who had recently returned from outside of Thailand testing positive for SARS-CoV-2. They included multiple genetic variants such as types GH, GR and O. In March 2020 the patients classified as imported cases—including returned travellers and the group who had undertaken a religious pilgrimage from the southern part of Thailand^23^—were identified as having type O. After the land border closure and suspension of all international flights, the number of cases decreased (Fig. 1). These interventions may help to limit imported cases. A new cohort of imported cases identified in May 2020 included a group of migrant workers in the southern part of Thailand^24^ with type G2 SARS-CoV-2. This indicated multiple introductions of SARS-CoV-2, and that there may be an outbreak in the southern part of Thailand.

In the current study no specific clinical signs were significantly associated with any specific SARS-CoV-2 types. Upper respiratory infection, fever, coughing, sore throat, and runny nose were the most common symptoms in COVID-19 patients, as has been frequently previously reported^25–28^. Clinical outcomes may associate with host factors such as age, lymphocytopenia, and cytokine responsiveness rather than SARS-CoV-2 genetic factors^29^. As of 22 May 2020 only one of the forty patients involved in the present study had been admitted to the ICU, and all had been discharged from hospital. In the 40 patients analysed in the present study the clinical course of COVID-19 was generally mild. The percentage of patients admitted to the ICU was 2.8%, the percentage with concurrent pneumonia was 22.2%, the percentage who were asymptomatic was 36.1%, and there were no fatalities, suggesting that SARS-CoV-2 does not usually lead to severe disease, unlike SARS-CoV and MERS^30,31^. These clinical data are similar to reports derived from China in which approximately 80% of confirmed cases were considered mild, 15% of confirmed cases were diagnosed as severe with pneumonia, and approximately 5% were deemed critical cases^32^.

The reported case-fatality rate of COVID-19 in Thailand (1.9%) is lower than that of SARS (22%)^30^, as it is in several countries including Italy (9.3%), Iran (7.8%), Spain (6.2%), the UK (4.9%), the Netherlands (4.3%), France (4.2%), China (4.0%), and the USA (1.3%)^33^. Reports suggest that elderly COVID-19 patients are at higher risk of hospitalization, pulmonary complications, and death^25,28,34^, as are elderly SARS and MERS patients^35,36^. The multiple origins of SARS-CoV-2 transmission into Thailand identified in the current study via phylogenetic analysis are similar to the pattern identified in Shanghai^29^.

Our study had some limitations. We did not analyse the whole-genome sequence for all samples due to time and cost. Therefore, we may have missed some genetic polymorphisms, which could potentially be of interest and reveal novel subclades to SARS-CoV-2. Due to the small sample size, a significant correlation between clinical symptoms and the viral genetic variation was not obtained. The samples we obtained encompassed the peak outbreak activity in Thailand at that time, so although they are limited in numbers, we believe that they may be sufficiently representative despite the relatively low sample size compared to other studies.

In summary, in the present study SARS-CoV-2 tracking and sites of origin were investigated in Thailand via genetic analysis. Most patients exhibited mild febrile illness without sequelae, but multiple origins of SARS-CoV-2 were evident. Understanding viral genetic and transmission patterns may facilitate more accurate prediction of future trends, and assist the development of more informed intervention policies.

## Supplementary information

Supplementary file1

Supplementary file2

Supplementary file3
