# Supplementary material for: Molecular epidemiology of the first wave of severe acute respiratory syndrome coronavirus 2 infection in Thailand in 2020
Source: Sci Rep. 2020 Oct 6;10:16602. doi: 10.1038/s41598-020-73554-7 (PMC7538975; doi:10.1038/s41598-020-73554-7)
Supplement: Supplementary file 1 — Supplementary file1 [file 41598_2020_73554_MOESM1_ESM.docx]

**Figure S1**. The location of the sample in this study. The specimens were collected from Bangkok (N=31), Nonthaburi (N=1), Samut Prakan (N=3), Songkla (N=1), Ubon Ratchathani (N=1), and Yala (N=1).
