# Supplementary figures and images for: Molecular epidemiology of the first wave of severe acute respiratory syndrome coronavirus 2 infection in Thailand in 2020
Source: Sci Rep. 2020 Oct 6;10:16602. doi: 10.1038/s41598-020-73554-7 (PMC7538975; doi:10.1038/s41598-020-73554-7)

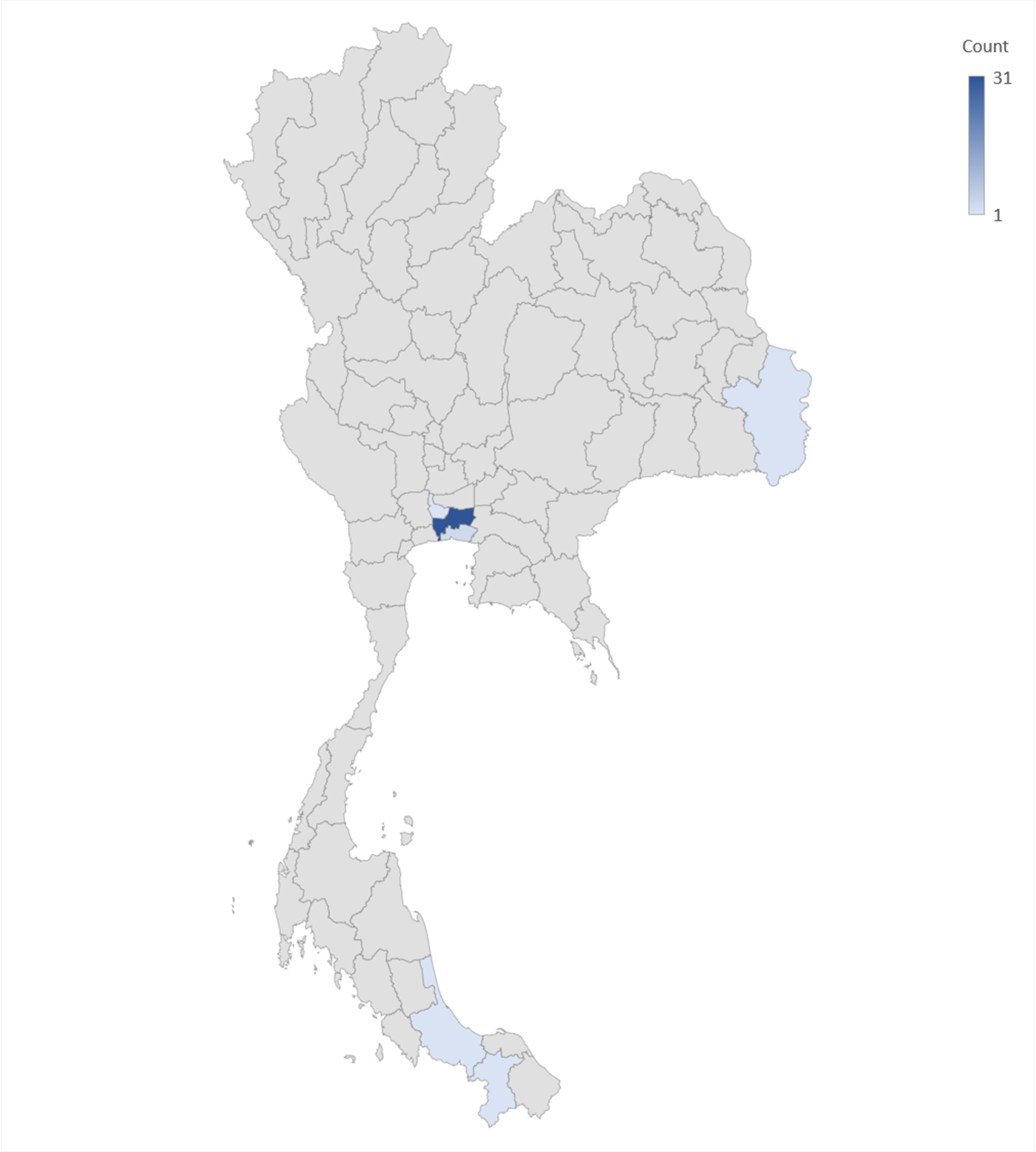

Supplement: Supplementary file 2 — Supplementary file2 [file 41598_2020_73554_MOESM2_ESM.tif]
