# Supplementary material for: Molecular epidemiology of the first wave of severe acute respiratory syndrome coronavirus 2 infection in Thailand in 2020
Source: Sci Rep. 2020 Oct 6;10:16602. doi: 10.1038/s41598-020-73554-7 (PMC7538975; doi:10.1038/s41598-020-73554-7)
Supplement: Supplementary file 3 — Supplementary file3 [file 41598_2020_73554_MOESM3_ESM.pdf]

Molecular epidemiology of the first wave of severe acute respiratory syndrome coronavirus 2 infection in Thailand in 2020

Jiratchaya Puenpa, Kamol Suwannakarn, Jira Chansaenroj, Pornjarim Nilyanimit, Ritthideach Yorsaeng, Chompoonut Auphimai, Rungrueng Kitphati, Anek Mungaomklang, Amornmas Kongklieng, Chintana Chirathaworn, Nasamon Wanlapakorn and Yong Poovorawan

Table S1 Primers used for conventional RT-PCR assays.

**Primer name**	**Nucleotide sequence 5´- 3´**	**Strand**	**Target gene**	**Product size (bp)**
N1_F28101	CCCATTCAGTACATCGATATCGG	Sense	N	744
N1_R28845	CGACTACGTGATGAGGAACGAG	Antisense		
N2_F28729	TAACAATGCTGCAATCGTGCTAC	Sense	N	960
N2_R29689	ACTGATTAAAGATTGCTATGTGAGA	Antisense		
S1_F21301	CCACGCGAACAAATAGATGGTTA	Sense	Spike	1030
S1_R22331	CTGAAGAAGAATCACCAGGAGTC	Antisense		
S2_F22188	CTATTAATTTAGTGCGTGATCTCC	Sense	Spike	1035
S2_R23223	TCAGTAAGAACACCTGTGCCTG	Antisense		
S3_F22978	CTATCAGGCCGGTAGCACACC	Sense	Spike	1118
S3_R24096	GCAATATCACCAAGGCAATCACC	Antisense		
S4_F23865	CTGGAATAGCTGTTGAACAAGAC	Sense	Spike	1083
S4_R24948	ACAACATCACAGTTACCAGACAC	Antisense		
S5_F24803	GAACTTCACAACTGCTCCTGCC	Sense	Spike	670
S5_R25472	TCTGAAGGAGTAGCATCCTTGA	Antisense		
E_F26147	CATCCGGAGTTGTTAATCCAGTA	Sense	E	440
E_R26589	CTAGGTTCCATTGTTCAAGGAGC	Antisense		
RdRp1_F13244	TGTCTGTACTGCCGTTGCCAC	Sense	RdRp	978
RdRp1_R14222	AAGTCAGTGTCAACATGTGACTC	Antisense		
RdRp2_F14115	CACGCCAGGTAGTGGAGTTCC	Sense	RdRp	1005
RdRp2_R15120	GACACCAGCTACGGTGCGAGC	Antisense		
RdRp3_F14997	GAGTTATGAGGATCAAGATGCAC	Sense	RdRp	1309
RdRp3_R16306	GTCTACGTATGCAAGCACCAC	Antisense		
86_F	TCAGGTGATGGCACAACAAGTC	Sense	Orf3a	412
86_R	ACGAAAGCAAGAAAAAGAAGTACGC	Antisense		
29_F	ACTTGTGTTCCTTTTTGTTGCTGC	Sense	Orf1a	387
29_R	AGTGTACTCTATAAGTTTTGATGGTGTGT	Antisense		

Table S2 GenBank accession numbers

		Accession numbers						Polymorphism position							
**No.**	**Code**	**SNP_orf1a**	**RdRp**	**S**	**E**	**N**	**Type**	**ORF1a**	**ORF1b**	**S**	**ORF8**	**ORF9b**	**ORF14**	**ORF14**	**ORF14**
1	WU5	MT502900	MT502940	MT502980	MT503020	MT503060	L								
2	WU14	MT502901	MT502941	MT502981	MT503021	MT503061	O		C13730T	C23929T		C28311T			
3	WU9	MT502902	MT502942	MT502982	MT503022	MT503062	S (T)	C8782T		G24047A	T28144C				
4	WU10	MT502903	MT502943	MT502983	MT503023	MT503063	S (T)	C8782T		G24047A	T28144C				
5	WU11	MT502904	MT502944	MT502984	MT503024	MT503064	S (T)	C8782T		G24047A	T28144C				
6	WU12	MT502905	MT502945	MT502985	MT503025	MT503065	S (T)	C8782T		G24047A	T28144C				
7	WU15	MT502906	MT502946	MT502986	MT503026	MT503066	S (T)	C8782T		G24047A	T28144C				
8	WU28	MT502907	MT502947	MT502987	MT503027	MT503067	S (T)	C8782T		G24047A	T28144C				
9	WU34	MT502908	MT502948	MT502988	MT503028	MT503068	S (T)	C8782T		G24047A	T28144C				
10	WU36	MT502909	MT502949	MT502989	MT503029	MT503069	S (T)	C8782T		G24047A	T28144C				
11	WU38	MT502910	MT502950	MT502990	MT503030	MT503070	S (T)	C8782T		G24047A	T28144C				
12	WU42	MT502911	MT502951	MT502991	MT503031	MT503071	S (T)	C8782T		G24047A	T28144C				
13	WU44	MT502912	MT502952	MT502992	MT503032	MT503072	S (T)	C8782T		G24047A	T28144C				
14	WU49	MT502913	MT502953	MT502993	MT503033	MT503073	S (T)	C8782T		G24047A	T28144C				
15	WU51	MT502914	MT502954	MT502994	MT503034	MT503074	S (T)	C8782T		G24047A	T28144C				
16	WU52	MT502915	MT502955	MT502995	MT503035	MT503075	G1		C14408T	A23403G					
17	WU53	MT502916	MT502956	MT502996	MT503036	MT503076	S (T)	C8782T		G24047A	T28144C				
18	WU54	MT502917	MT502957	MT502997	MT503037	MT503077	S (T)	C8782T		G24047A	T28144C				
19	WU56	MT502918	MT502958	MT502998	MT503038	MT503078	S (T)	C8782T		G24047A	T28144C				
20	WU60	MT502919	MT502959	MT502999	MT503039	MT503079	S (T)	C8782T		G24047A	T28144C				
21	WU63	MT502920	MT502960	MT503000	MT503040	MT503080	S (T)	C8782T		G24047A	T28144C				
22	WU69	MT502921	MT502961	MT503001	MT503041	MT503081	S (T)	C8782T		G24047A	T28144C				
23	WU70	MT502922	MT502962	MT503002	MT503042	MT503082	S (T)	C8782T		G24047A	T28144C				
24	WU72	MT502923	MT502963	MT503003	MT503043	MT503083	S (T)	C8782T		G24047A	T28144C				
25	WU73	MT502924	MT502964	MT503004	MT503044	MT503084	G1		C14408T	A23403G					
26	WU117	MT502925	MT502965	MT503005	MT503045	MT503085	G2		C14408T	A23403G			G28811A	G28812A	G28813C

27	WU114	MT502926	MT502966	MT503006	MT503046	MT503086	G1		C14408T	A23403G					
28	WU98	MT502927	MT502967	MT503007	MT503047	MT503087	S (T)	C8782T		G24047A	T28144C				
29	WU31	MT502928	MT502968	MT503008	MT503048	MT503088	S (T)	C8782T		G24047A	T28144C				
30	WU113	MT502929	MT502969	MT503009	MT503049	MT503089	G2		C14408T	A23403G			G28811A	G28812A	G28813C
31	WU68	MT502930	MT502970	MT503010	MT503050	MT503090	S (T)	C8782T		G24047A	T28144C				
32	WU41	MT502931	MT502971	MT503011	MT503051	MT503091	S (T)	C8782T		G24047A	T28144C				
33	WU118	MT502932	MT502972	MT503012	MT503052	MT503092	S (T)	C8782T		G24047A	T28144C				
34	WU135	MT502933	MT502973	MT503013	MT503053	MT503093	G1		C14408T	A23403G					
35	WU125	MT502934	MT502974	MT503014	MT503054	MT503094	S (T)	C8782T		G24047A	T28144C				
36	WU129	MT502935	MT502975	MT503015	MT503055	MT503095	G1		C14408T	A23403G					
37	WU130	MT502936	MT502976	MT503016	MT503056	MT503096	G1		C14408T	A23403G					
38	WU111	MT502937	MT502977	MT503017	MT503057	MT503097	G2		C14408T	A23403G			G28811A	G28812A	G28813C
39	WU187	MT502938	MT502978	MT503018	MT503058	MT503098	G1		C14408T	A23403G					
40	WU138	MT502939	MT502979	MT503019	MT503059	MT503099	O		C13730T	C23929T		C28311T			
